# Bacteriocins attenuate *Listeria monocytogenes–*induced intestinal barrier dysfunction and inflammatory response

**DOI:** 10.1007/s00253-024-13228-w

**Published:** 2024-06-19

**Authors:** Zhao Wang, Jing Du, Wenyu Ma, Xinjie Diao, Qi Liu, Guorong Liu

**Affiliations:** 1 School of Food and Health, Beijing Technology and Business University, Beijing, 100048 China; 2 Key Laboratory of Geriatric Nutrition and Health, Ministry of Education, Beijing Technology and Business University, Beijing, 100048 China; 3 Beijing Engineering and Technology Research Center of Food Additives, Beijing Technology and Business University, No. 11 Fucheng Road, Haidian District, Beijing, 100048 China

**Keywords:** Bacteriocin, Antibacterial activity, Inflammatory responses, Intestinal epithelial barrier, Virulence factors

## Abstract

**Abstract:**

Bacteriocins have the potential to effectively improve food-borne infections or gastrointestinal diseases and hold promise as viable alternatives to antibiotics. This study aimed to explore the antibacterial activity of three bacteriocins (nisin, enterocin Gr17, and plantaricin RX-8) and their ability to attenuate intestinal barrier dysfunction and inflammatory responses induced by *Listeria monocytogenes*, respectively. Bacteriocins have shown excellent antibacterial activity against *L. monocytogenes* without causing any cytotoxicity. Bacteriocins inhibited the adhesion and invasion of *L. monocytogenes* on Caco-2 cells, lactate dehydrogenase (LDH), trans-epithelial electrical resistance (TEER), and cell migration showed that bacteriocin improved the permeability of Caco-2 cells. These results were attributed to the promotion of tight junction proteins (TJP) assembly, specifically zonula occludens-1 (ZO-1), occludin, and claudin-1. Furthermore, bacteriocins could alleviate inflammation by inhibiting the mitogen-activated protein kinase (MAPK) and nuclear factor kappa B (NF-κB) pathways and reducing the secretion of interleukin-6 (IL-6), interleukin-1 β (IL-1β) and tumor necrosis factor α (TNF-α). Among three bacteriocins, plantaricin RX-8 showed the best antibacterial activity against *L. monocytogenes* and the most pronounced protective effect on the intestinal barrier due to its unique structure. Based on our findings, we hypothesized that bacteriocins may inhibit the adhesion and invasion of *L. monocytogenes* by competing adhesion sites. Moreover, they may further enhance intestinal barrier function by inhibiting the expression of *L. monocytogenes* virulence factors, increasing the expression of TJP and decreasing the secretion of inflammatory factors. Therefore, bacteriocins will hopefully be an effective alternative to antibiotics, and this study provides valuable insights into food safety concerns.

**Key points:**

*• Bacteriocins show excellent antibacterial activity against L. monocytogenes*

*• Bacteriocins improve intestinal barrier damage and inflammatory response*

*• Plantaricin RX-8 has the best protective effect on Caco-2 cells damage*

**Graphical Abstract:**

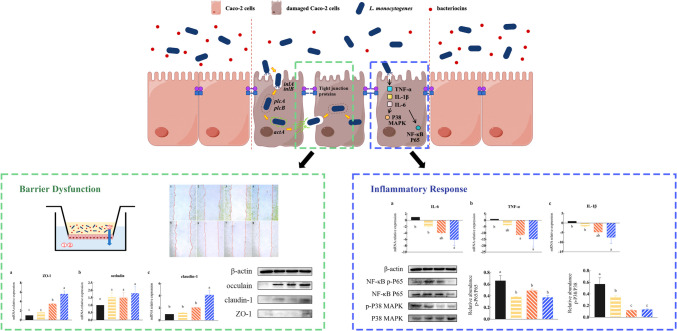

**Supplementary Information:**

The online version contains supplementary material available at 10.1007/s00253-024-13228-w.

## Introduction

Food-borne pathogens pose a significant risk to food safety and public health, causing bacterial infections and food poisoning, which places a huge burden on public healthcare. It has been reported that the incidence of food-borne diseases ranks second among the incidence of all types of diseases (Bintsis 2017). There is increasing evidence that food-borne pathogens such as *Salmonella*, *Escherichia coli*, and *Listeria monocytogenes* cause food poisoning and even death by entering the human body through their contaminated food (Kumariya et al. 2019). Generally, antibiotics are often considered to be one of the most effective interventions in human medicine. While antibiotics can control inflammation to a certain extent, there are potential side effects of drug-acquired resistance, allergic reactions, and toxic reactions that would limit their wide use in clinical applications. Alternative strategies to control food-borne pathogens are of paramount importance.

Bacteriocin, a naturally occurring antibacterial polypeptide produced by lactic acid bacteria, was considered to be a promising antibacterial agent due to its high specificity, low toxicity, and no resistance. These appealing advantages of bacteriocins have strongly motivated research into their application in inhibiting pathogens. Zhang et al. (2023) have shown that the novel bacteriocin RSQ01 of *Lactococcus lactis* exhibited an extensive antimicrobial activity against both gram-positive and gram-negative bacteria, which can be applied in milk preservation. Additionally, Xin et al. (2023) discovered that the bacteriocin LFX01 has antibacterial activity against *Staphylococcus aureus* and *E.coli*, could be a potential preservative for pork. The antibacterial activity of bacteriocins is related to their primary structure, physical and chemical characteristics, and antibacterial mechanism, which can be classified into four classes (I–IV). Class I bacteriocins are post-translational modified peptides that contain mainly lanolin sulfur amino acids, such as lanthionine and β-methyllanthionine. The class I bacteriocins comprise three subgroups: class Ia (lantibiotics), class Ib (labyrinthopathies), and class Ic (sanctibiotics), which interact with negatively charged membranes to generate pores and are especially effective against gram-positive bacteria (Lubelski et al. 2008). Class II bacteriocins do not have lanolin amino acid residues and comprise four subgroups: class IIa (pediocin-like bacteriocins), class IIb (two-peptide bacteriocins), class IIc (circular bacteriocins), and class IId (linear and non-pediocin-like bacteriocins). Specifically, the class IIa bacteriocins contain the conserved YGNGVXC sequence at the N-terminus, which can effectively inhibit *L. monocytogenes* effectively (Ennahar et al. 2000). The structure formed by the peptide chain of class IIb bacteriocins binds to the cell wall, forming a pore that leads to bacteria (Shai 1999). The class III bacteriocins are macromolecular protein bacteriocins that include colicins, helveticin M, helveticin J, and enterolysin A (Abriouel et al. 2011; Verma et al. 2022). Class IV bacteriocin complex contains lipids and carbohydrate moieties. As research has deepened, it has found that the structure of bacteriocins has a crucial impact on their physiological function. Plantaricin FB-2 (class IIb bacteriocin), produced by *Lactiplantibacillus plantarum*, exerts its antibacterial ability by disrupting the structure of the bacterial cell membrane, which increases cell permeability and results in the leakage of cytoplasmic contents (Li et al. 2023). While class IIa bacteriocins primarily penetrate susceptible microbial membranes, probably by forming pore complexes, causing anionic imbalance and inorganic phosphate leakage (Ennahar et al. 2000). Thus, the different structures of bacteriocins lead to different bacteriostatic mechanisms and bacteriostatic effects.

Previous studies have indicated that bacteriocins potentially inhibit or kill drug-resistant pathogens and actively regulate the gut microbiota (Yi et al. 2018; Yu et al. 2023). The intestinal barrier serves as a crucial target through which bacteriocins exert their intestinal health effects. When the intestinal barrier is disrupted, inflammation occurs, immune cells are stimulated, and inflammatory factors are activated. These adverse effects lead to the breakdown of the intestinal epithelial tight-junction structure with the cells of the gut, increasing intestinal permeability, facilitating the entry of pathogens, toxins, and other substances into the body, ultimately leading to the occurrence of disease. Research has indicated that when pathogenic bacteria infiltrate cells, bacteriocins could protect the cell barrier (Yu et al. 2018). It was reported that the biogenic MccJ25 can raise the trans-epithelial electrical resistance (TEER), decrease the lactate dehydrogenase (LDH), and promote tight junction proteins (TJP), thereby protecting against intestinal damage and the inflammatory response induced by ETEC K88. Until now, there has been sufficient evidence to validate the antibacterial activity of bacteriocins against food-borne pathogenic bacteria in vitro. However, the mechanisms by which different bacteriocins protect the intestinal barrier function during the invasion by food-borne pathogenic bacteria and their bacteriostatic effects need further investigation. Further research is needed to elucidate the mechanisms by which bacteriocins defend the intestinal barrier against invasion by food-borne pathogenic bacteria. This will aid in combating intestinal barrier dysfunction induced by virulence factors involved in pathogenic bacterial invasion.

To fulfill this demand, this study aimed to evaluate the in vitro antibacterial activity of three bacteriocins (nisin, enterocin Gr17, and plantaricin RX-8) and their ability to attenuate intestinal barrier dysfunction and inflammatory responses induced by *L. monocytogenes*, respectively. First, the antibacterial activity and toxicity of the three bacteriocins were investigated. In addition, the protective effect of bacteriocins on intestinal barrier function during the invasion of *L. monocytogenes* was evaluated in terms of anti-adhesion properties, virulence factor expression, intestinal epithelial permeability, tight junction structure, and inflammatory factor secretion. This study provides a theoretical basis for future research on the use of bacteriocins to prevent pathogenic bacteria in vivo.

## Materials and methods

### Bacterial strains and growth conditions

The enterocin Gr17 (class IIa bacteriocin) and plantaricin RX-8 (class IIb bacteriocin) were produced by *Enterococcus faecalis* Gr17 (CGMCC 16677) and *Lactiplantibacillus paraplantarum* RX-8 (CGMCC 20852), as described in a previous study (Liu et al. 2021; Liu et al. 2019). Nisin was purchased from LuQiao (Beijing, China). *L. monocytogenes* is a common gram-positive foodborne pathogen with strong pathogenicity. Thus, *L. monocytogenes* (ATCC 35152) was used as an indicator strain for evaluating the antibacterial activity. *E. faecium* Gr17 and *L. paraplantarum* RX-8 were cultured in De Man, Rogosa and Sharpe Broth (MRS, LuQiao, Beijing, China) at 37 °C. *L. monocytogenes* was grown in Trypticase Soy Broth (TSB, LuQiao, Beijing, China) at 37 °C.

### Purification of bacteriocins

The purification of bacteriocins were performed by a two-step method (Arifin et al. 2021). Briefly, *E. faecium* Gr17 and *L. paraplantarum* RX-8 were cultured to the logarithmic growth phase and inoculated at 2% inoculum in MRS broth for 24 h at 37 °C. Then, supernatants were collected by centrifugation (4 °C, 10,000 rpm/min, 10 min) (Beckman Coulter, CA, USA). A 10-fold concentration was generated by a vacuum freezing concentrator (Jiaimu, Beijing, China).

The bacteriocin crude extract of enterocin Gr17 and plantaricin RX-8 were purified by GE AKTA pure system (General Electric, NY, USA) equipped with a HiPrep SP Sepharose FF 16/10 column. Elution was performed using a buffer solution of 0.02 M citric acid-sodium citrate and 1 M sodium chloride. The following GE AKTA purity parameters were employed: sample loading, 1 mL; flow rate, 0.5 mL/min. The obtained samples were freeze-dried and stored at −80 °C.

### Determination of the minimum inhibitory concentration

The minimum inhibitory concentration (MIC) of enterocin Gr17, plantaricin RX-8, and nisin were assessed using microdilution assays (Lin et al. 2022). In brief, the three bacteriocins were dissolved with phosphate buffered saline (PBS), and two-fold serial dilutions of bacteriocins were added into 96-well microplates (Corning, NY, USA). The concentrations of three bacteriocins ranged from 0.5 to 128 μg/mL. Fifty microliters of *L. monocytogenes* (1 × 10^8^ CFU/mL) was inoculated into each well. After a 24-h incubation at 37 °C, the optical density at 600 nm was recorded utilizing an automated microplate reader.

### Assays of antibacterial activity

The antibacterial activity of three bacteriocins were assessed in accordance with the specified protocol, with some modifications (Gao et al. 2023). The *L. monocytogenes* (1 × 10^8^ CFU/mL) was added to three bacteriocins (0, 1/4, 1/2, 1 ×MIC) (*v*/*v* =1/1) for 0–4 h at 37 °C, respectively. Bacteria counts were assessed on Tryptic Soy Agar (TSA) plates and logarithm of CFU/mL was plotted against time to determine the in vitro antibacterial activity of bacteriocins.

### Cell culture

Human colorectal adenocarcinoma cells (Caco-2 cells, ATCC ATB-37) were obtained from the American Type Culture Collection and cultured in Dulbecco's Modified Eagle Medium (DMEM, Gibco, CA, USA) supplemented with 20% (v/v) fetal bovine serum, 1% streptomycin (10,000 g/mL)/penicillin (10,000 U/mL) and 1% non-essential amino acids under a 95% humidified atmosphere of 5% CO_2_ at 37 °C with media replacement every 2 days.

### Assays of cytotoxicity

Caco-2 cells were seeded at a density of 2 × 10^5^ cells/mL in 96-well plates. The cells were treated with bacteriocin solution at concentrations of 0, 1/4, 1/2 and 1 ×MIC for 2 h to 80% confluence. Wells containing untreated cells were used as controls. Finally, cell viability was determined using Cell Counting Kit-8 (CCK-8, Solarbio, Beijing, China), and the data were expressed as a percentage of the control.

### Inhibition of *L. monocytogenes* adhesion to Caco-2 cells by bacteriocins

The adhesion inhibitory effect was determined according to the method of Guo et al.’s (2022) with slight alterations. The adhesion inhibitory effect of *L. monocytogenes* on Caco-2 cells was assessed by competition, exclusion, and displacement experiments using three bacteriocins. For the competition assay, three bacteriocins and *L. monocytogenes* were co-incubated with Caco-2 cells at 37 °C for 4 h, respectively. For the exclusion experiment, bacteriocin was inoculated into Caco-2 cells for 2 h, followed by 2 h of *L. monocytogenes*. For the displacement experiment, *L. monocytogenes* was inoculated into Caco-2 cells for 2 h before bacteriocin was added. In three assays, cell cultures were flushed with PBS to eliminate non-adherent bacteria and then lysed with 1 mL of Triton-100 solution (0.1% v/v in PBS). After 10 min of incubation at 37 °C, the solution with released bacteria cells was serially diluted and plated on PALCAM agar. The plates were incubated at 37 °C for 48 h. Adhesion inhibition rate was determined by comparing the adhesion of *L. monocytogenes* in the presence of bacteriocins to that of *L. monocytogenes* alone. The control group contained only the *L. monocytogenes* suspensions.

### Inhibition of *L. monocytogenes* invasion to Caco-2 cells by bacteriocins

The inhibitory effect of three bacteriocins on *L. monocytogenes* invading Caco-2 cells by *L. monocytogenes* was estimated using the colony counts and virulence factor gene expression levels of *L. monocytogenes*-based assay. The methods described by Arvaniti et al. (2023) were used with some modifications. Exclusion experiments were used to evaluate the invasion assay. On the basis of adhesion experiments, Caco-2 cells were incubated with gentamicin (200 μg/mL) for 1 h to eliminate non-invading adherent *L. monocytogenes*. Subsequently, the invasion effect of Caco-2 cells by *L. monocytogenes* was evaluated by the same treatment and counting method as in the adhesion experiment. The above experiments were performed to determine the colony counts of *L. monocytogenes* at 0–4 h, respectively. The control group contained only *L. monocytogenes* suspensions without adding bacteriocin.

### Evaluation of the permeability of the Caco-2 cells monolayer

The permeability of Caco-2 cells monolayer was assessed according to the method of Yu et al. (2018) with some modifications. To explore the protective effect of the three bacteriocins against membrane damage induced by *L. monocytogenes* in Caco-2 cells, the integrity of Caco-2 cell monolayer was measured by evaluating the LDH activity and TEER. Specifically, Caco-2 cells (2 × 10^5^ cells/mL) were seeded into a 12-well transwell filter and cultured until confluent. Three bacteriocins and *L. monocytogenes* were added separately to 12-well transwell, and the TEER was measured after 24 h of co-culture with Caco-2 cells. The amount of *L. monocytogenes* and LDH content were measured by collecting the fluid in a subplate of transwell. The control group contained only *L. monocytogenes* suspension without adding bacteriocin.

### Evaluation of Caco-2 cells monolayer migration

The effect of cell migration was measured by cell scratch assay, transwell assay, and colony count determination. Caco-2 cells (2 × 10^5^ cells/mL) were plated on 24-well transwell filter and grown to confluence. The cells were intentionally injured by scratching to create a wound with an initial width of 1 mm. Subsequently, 2 mL of serum-free DMEM suspension containing three bacteriocins and *L. monocytogenes* (*v*/*v* = 1/1) was added to the well, respectively. After incubating for 4 h, the distance covered by cell migration was measured. To quantify the scratches, the area and width were measured using the ImageJ software. Concurrently, the number of cells traversing through the membrane in transwell plate was measured.

### RT-qPCR assay

The gene expression levels of virulence factors reflect the whole process of invasion of Caco-2 cells by *L. monocytogenes*; the gene expression levels of pro-inflammatory factors tumor necrosis (TNF-α), interleukin-6 (IL-6), and interleukin-1 beta (IL-1β) represent the inflammation severity; TJP gene expression levels zonula occludens-1 (ZO-1), occludin, and claudin-1 represent the integrity of intestinal epithelial barrier, all of which can be evaluated via quantitative real-time PCR (RT-qPCR) (Gao et al. 2023).

According to the manufacturer’s instructions, the RNAprep Pure Cell/Bacteria Kit (DP430, Tiangen, Beijing, China) was used. The FastKing gDNA Dispelling RT SuperMix (FP314, Tiangen, Beijing, China) was used to synthesize total RNA from cDNA. An Applied Biosystems 7500 real-time PCR system (Applied Biosystems, CA, USA) was used to amplify cDNA using primers (Table S1). The β-actin protein was utilized as an endogenous control.

### Western blot assay

Western blotting can be employed to assess TJP expression levels associated with intestinal barrier function and key proteins involved in the immune pathways. To estimate protein expression associated with TJP and inflammation pathways, the method described by Arvaniti et al. (2023) was followed. Caco-2 cells (2.0 × 10^5^ cells/mL) were added to the 6-well plate before flushing with PBS. Proteins were extracted using radioimmunoprecipitation buffer (RIPA, Solarbio, Beijing, China), and its concentration was measured with Bicinchoninic acid kit (BCA, Solarbio, Beijing, China). Equal amounts of proteins from each sample were separated by 12% sodium dodecyl sulfate-polyacrylamide gel electrophoresis (SDS-PAGE). The membranes were blocked with 5% skimmed milk for 2 h. Antibodies of ZO-1, occludin, and claudin-1, P38 mitogen-activated protein kinase (P38 MAPK), phosphorylated P38 MAPK (p-P38 MAPK), nuclear factor kappa B P65 (NF-κB P65), phosphorylated NF-κB P65 (NF-κB p-P65), and β-actin (1:1000) were incubated with membranes overnight at 4 °C. After incubation, membranes were washed five times using tris-buffered saline with Tween 20 (TBST) and incubated with appropriate horseradish peroxidase-labelled secondary antibodies (1:1000, Beyotime Biotechnology, Shanghai, China) at 37 °C for 0.5 h. Following washing with TBST, protein bands were visualized using Fluor Chem imaging system (Alpha Innotech, CA, USA) and band intensities were quantified using ImageJ software. To calculate relative protein expression, the relative concentration of β-actin was used as an internal control.

### Statistical analysis

All the experiments were performed in triplicate. The results were analyzed by variance (ANOVA) and Duncan’s test with SPSS 23.0 software (SPSS, IL, USA). All the data were visualized using GraphPad Prism 6 software. The results were presented as *mean* ± *standard deviation* (*SD*). *P* < 0.001 indicated statistical significance.

## Results

### *In vitro* antimicrobial ability of bacteriocins

Firstly, the potencies of nisin, enterocin Gr17, and plantaricin RX-8 were harmonized by agar diffusion method, and the standardized concentration was 128 μg/mL (Fig. S1). The antimicrobial activity of three bacteriocins (nisin, enterocin Gr17, and plantaricin RX-8) against *L. monocytogenes* was evaluated with MIC assay. They all demonstrated highly effective in inhibiting *L. monocytogenes* at an MIC of 16 μg/mL (Table S2).

**Fig. 1:**
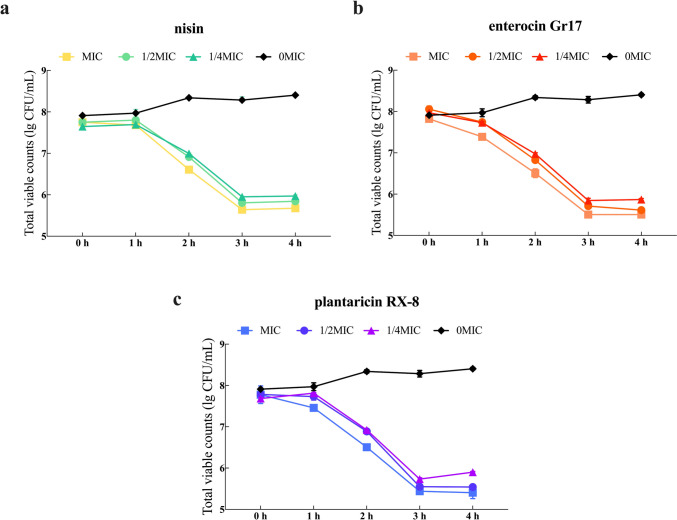
Antibacterial activity of nisin (**a**), enterocin Gr17 (**b**) and plantaricin RX-8 (**c**) against *L. monocytogenes in vitro*

Fig. 1a, b, and c show that nisin, enterocin Gr17, and plantaricin RX-8 at the concentrations of 1 ×MIC, 1/2 ×MIC, and 1/4 ×MIC all inhibit the growth of *L. monocytogenes,* respectively. Among them, the antimicrobial ability of bacteriocins at 1 MIC against *L. monocytogenes* were significantly greater than that of other concentrations (*P* < 0.001). Furthermore, all three bacteriocins started to exert antibacterial effects from 1 h and reached their optimal antibacterial activity at 3 h, after which the bacteriocins still maintain their activity at 4 h.

### Cytotoxicity studies of bacteriocins in Caco-2 cells

**Fig. 2:**
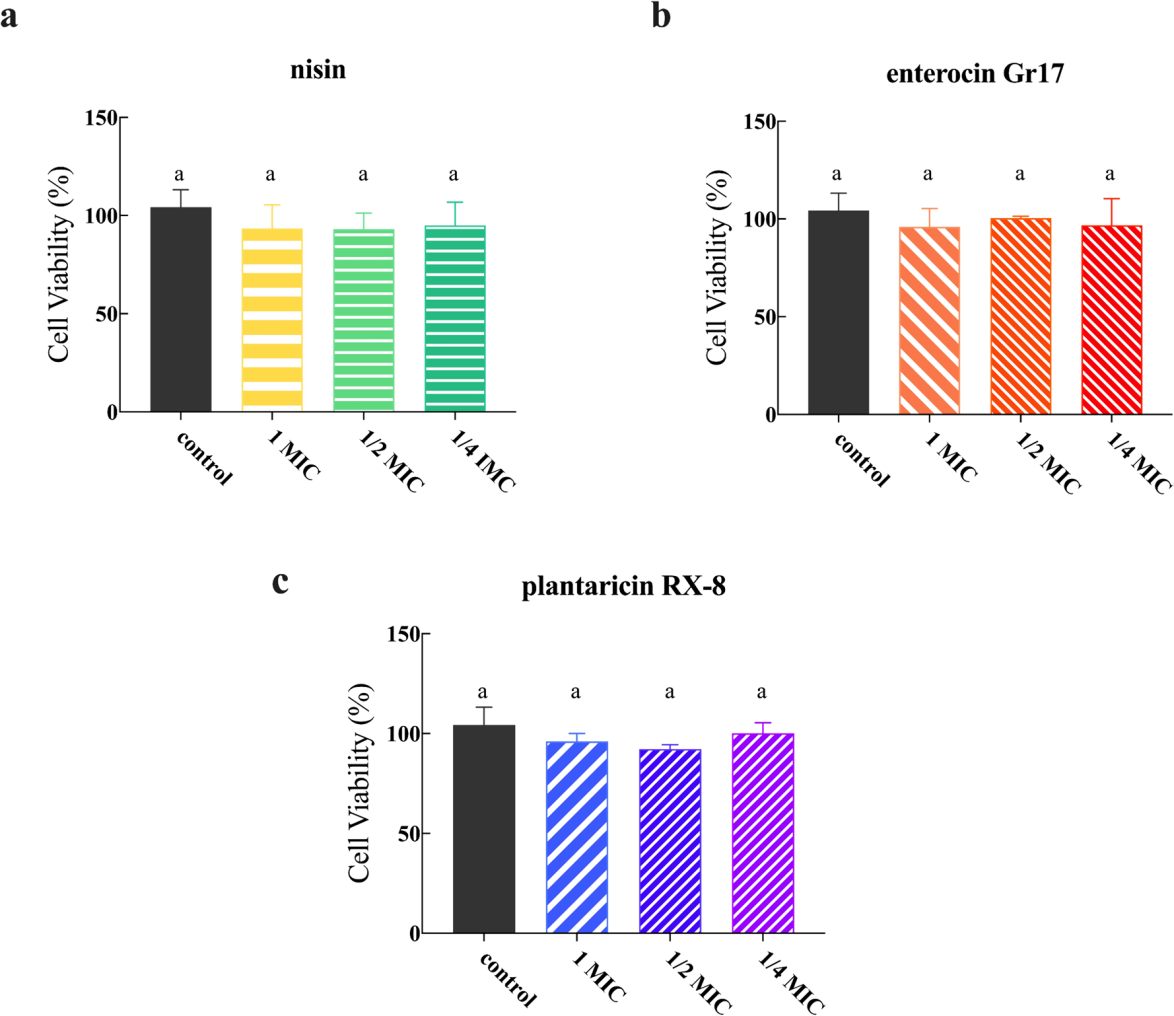
Cell cytotoxicity assays of nisin (**a**), enterocin Gr17 (**b**) and plantaricin RX-8 (**c**) in Caco-2 cells

To assess the safety of the three bacteriocins, cytotoxicity assays were performed. The cell viability of bacteriocins (nisin, enterocin Gr17, and plantaricin RX-8) at 1 × MIC, 1/2 × MIC, and 1/4 × MIC was determined using a CCK-8 assay. As shown in Fig. 2a, b, and c, there are no significant differences in all results (*P* < 0.001), even though the concentrations of three bacteriocins are up to 1 × MIC. The results indicated that three bacteriocins had no cytotoxicity even at concentrations of 1 × MIC values against Caco-2 cells.

### Inhibitory effect of bacteriocins on adhesion of *L. monocytogenes* to Caco-2 cells

**Fig. 3:**
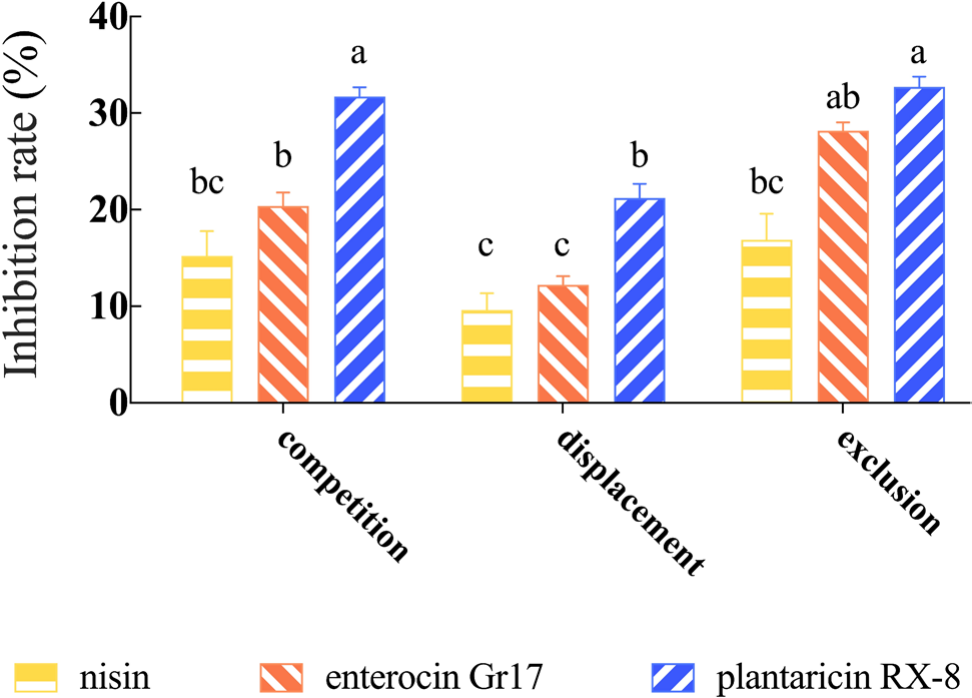
Protective effects of bacteriocins against *L. monocytogenes* adhesion Caco-2 cells. Competition (a), displacement (b) and exclusion (c)

To assess the effectiveness of bacteriocins in preventing adhesion of *L. monocytogenes* to Caco-2 cell monolayers, *in vitro* adhesion assays were conducted. In Fig. 3, the effect of three bacteriocins on *L. monocytogenes* adhesion to Caco-2 cells is illustrated under three action modes: competition, exclusion, or displacement. All three bacteriocins inhibited the adhesion of *L. monocytogenes* to Caco-2 cells. Compared to the control group, the treatment groups showed significantly lower total viable *L. monocytogenes* counts before and after intervention (*P* < 0.001). Comparing exclusion, competition, and displacement experiments, the number of *L. monocytogenes* in the bacteriocin displacement intervention was significantly higher than in the other action modes (*P* < 0.001). These results all demonstrated that bacteriocins inhibit the adhesion of *L. monocytogenes* to Caco-2 cells by competing with *L. monocytogenes* for binding sites (Gomes et al. 2012), suggesting that their mechanism of action involves interacting with the pathogen. Thus, exclusion experiments were performed to evaluate the effect of bacteriocins on the adhesion *L. monocytogenes* in Caco-2 cells. Furthermore, among three bacteriocins, plantaricin RX-8 had the best bacteriostatic effect. In the exclusion experiment, the total viable count of plantaricin RX-8 (5.05 lg CFU/mL) was significantly lower than that in nisin (6.29 lg CFU/mL) and enterocin Gr17 (5.49 lg CFU/mL).

### Inhibitory effects of bacteriocins on *L. monocytogenes* invasion of Caco-2 cells

Due to the fact that some *L. monocytogenes* can adhere to the surface of Caco-2 cells and continue to cause damage, the invasion of Caco-2 by *L. monocytogenes* during this process was investigated to evaluate the effectiveness of bacteriocins. Compared with the control group, three bacteriocins all inhibited the growth of *L. monocytogenes* both extracellularly and intracellularly in Caco-2 cells (Fig. S2). The antibacterial activities of three bacteriocins in extracellular were higher than those in intracellular. Interestingly, the antibacterial abilities of nisin, enterocin Gr17, and plantaricin RX-8 on the extracellular were similar, with a 1–2 lg CFU/mL reduction in the count of *L. monocytogenes* (Fig. S2a)*.* While plantaricin RX-8 and enterocin Gr17 inhibited *L. monocytogenes*, they were significantly better than nisin at the intracellular level (*P* < 0.001) (Fig. S2b). In particular, plantaricin RX-8 reduced the counts of *L. monocytogenes* adhering to Caco-2 cells reduced by 3 lg CFU/mL. In addition, compared with the inhibition evaluation *in vitro*, all the bacteriocins reduced *L. monocytogenes* by more than 2 lg CFU/mL *in vitro*, but only 1–2 lg CFU/mL in Caco-2 cells (Fig. S2c).

**Fig. 4:**
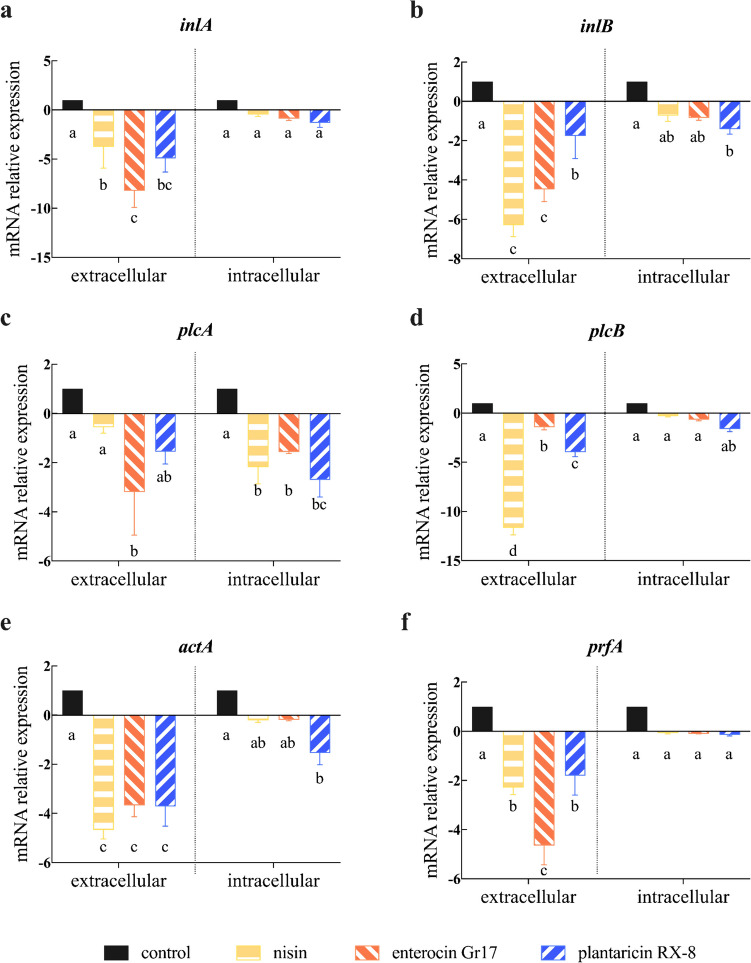
Changes in *L. monocytogenes* virulence factor gene expression *inlA* (**a**), *inlB* (**b**), *plcA* (**c**), *plcB* (**d**), *actA* (**e**) and *prfA* (**f**)

To further explore the inhibitory effect of bacteriocin on the invasion of Caco-2 cells by *L. monocytogenes*, an RT-qPCR assay was employed. The aim of experiments was to evaluate whether bacteriocins could inhibit the effect of *L. monocytogenes* on Caco-2 cells by altering the expression of associated virulence factors on *L. monocytogenes*, as well as to identify the site at which bacteriocins act. Specifically, *internalin A* (*inlA*), *internalin B* (*inlB*), *phospholipase A* (*plcA*), *phospholipase B* (*plcB*), *actin assembly-inducing protein A* (*actA*), and *positive regulatory factor A* (*prfA*) were the associated virulence factors whose corresponding gene expression levels were measured by RT-qPCR (Fig. 4). *InlA* and *inlB* assist in the entry of *L. monocytogenes* into cells (Arvaniti et al. 2023). Compared with those in the control group, the expression of all three bacteriocins was downregulated in the *L. monocytogenes* group. The transcription levels of extracellular inhibitory genes *inlA* and *inlB* were significantly greater than those of intracellular inhibitory genes (*P* < 0.001) (Fig. 4a, b). Furthermore, compared with the *inlB* gene, the *inlA* gene was significantly downregulated in the presence of bacteriocins in the extracellular (*P* < 0.001). Enterocin Gr17 and nisin had more pronounced effects on the *inlA* and *inlB* genes at extracellular, respectively, but plantaricin RX-8 significantly downregulated the gene expression of both the *inlA* and *inlB* genes at intracellular (*P* < 0.001). This may be related to the fact that different classes of bacteriocins have different mechanisms of antibacterial activity. These results imply that more plantaricin RX-8 may enter Caco-2 cells, where it can exert antibacterial activity intracellularly.

The relative level of virulence factors *plcA* (Fig. 4c) and *plcB* (Fig. 4d) were downregulated both intracellular and extracellular. The enterocin Gr17 was more effective against extracellular *plcA*, nisin more effective against for extracellular *plcB*, whereas plantaricin RX-8 affected *plcA* and *plcB* at intracellular, resulting in significant downregulation of virulence factor gene expression (*P* < 0.001). The *plcA* and *plcB* can dissolve vacuoles and help *L. monocytogenes* enter the cytoplasm (Kanki et al. 2018). Compared to the adhesion rate of *L. monocytogenes*, the results showed that bacteriocins are more important to the ability of *L. monocytogenes* intracellular. Thus, the antibacterial activity of plantaricin RX-8 was superior to the other two bacteriocins.

The migratory ability of *L. monocytogenes* was attributed to *actA*, which facilitates spread between cells (Rios-Covian et al. 2018). There were no significant differences in the antibacterial activity of the three bacteriocins on the basis of extracellular *actA* function (*P* < 0.001). However, among three bacteriocins, the relative expression level of *actA* in plantaricin RX-8 was significantly downregulated in intracellular (*P* < 0.001) (Fig. 4e). These results indicated that bacteriocin can exert antibacterial activity by inhibiting the intracellular acquisition of *L. monocytogenes*. Among them, the plantaricin RX-8 entered more Caco-2 cells and had the most pronounced effect.

The virulence factors *inlA*, *inlB*, *plcA*, *plcB*, and *actA* of *L. monocytogenes* are regulated by *prfA* (Rios-Covian et al. 2018). The relative expression level of the *prfA* gene in *L. monocytogenes* was downregulated both extracellularly and intracellularly in three bacteriocins, compared with the control group (Fig. 4f). Enterocin Gr17 significantly downregulated *prfA* expression at extracellular (*P* < 0.001), which was consistent with the inhibitory effect of *inlA* and *plcA*. These results suggested that enterocin Gr17, plantaricin RX-8, and nisin were effective in inhibiting the whole process of *L. monocytogenes* entry into Caco-2 cells, which included adhesion, invasion, entry into the cytoplasm, and intercellular propagation. Notably, plantaricin RX-8 can significantly reduce the entry of *L. monocytogenes* into cells and reduce the spread of *L. monocytogenes* in the cytoplasm and between cells. Therefore, plantaricin RX-8 exhibited a stronger activity against *L. monocytogenes* after internalization by Caco-2 cells, these results were consistent with the previous findings on the effect of bacteriocins on inhibiting *L. monocytogenes* invasion of Caco-2 cells.

### Effects of bacteriocins on *L. monocytogenes*-induced cellular permeability

**Fig. 5:**
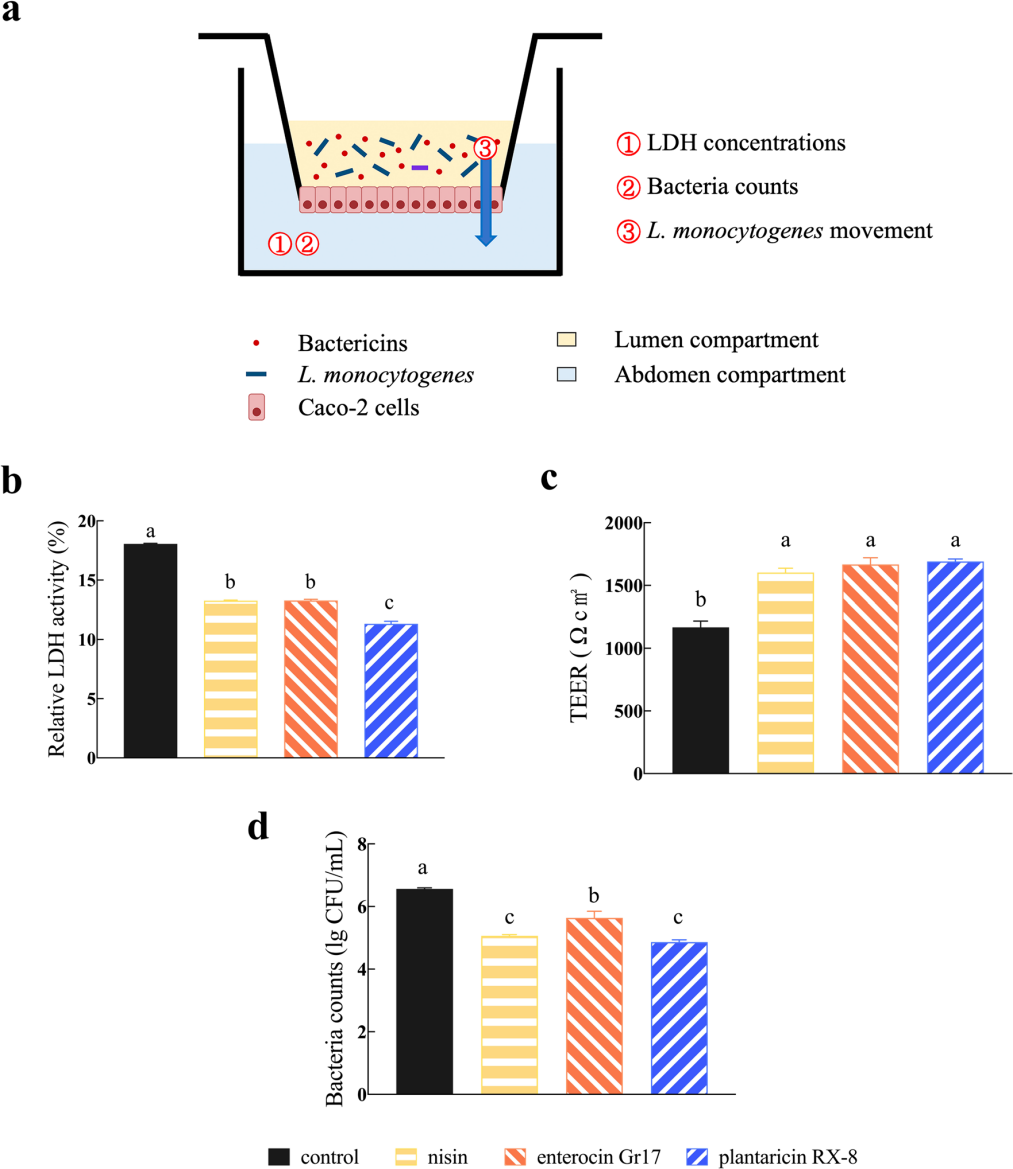
Evaluation of bacteriocins on *L. monocytogenes*-induced Caco-2 cells permeability. Schematic representation of the intestinal epithelium model (**a**), LDH activity (**b**), TEER (**c**) and the counts of *L. monocytogenes* (**d**)

To study the protective effect of bacteriocins against *L. monocytogenes*-induced membrane damage, the LDH activity, TEER, and the colony count of *L. monocytogenes* of the lower chamber were measured (Yu et al. 2018). As shown in Fig. 5a, a monolayer of Caco-2 cells is formed on the transwell membrane, and the upper and lower compartments are divided into the lumen compartment and abdomen compartment, respectively. As expected, the addition of bacteriocins significantly reduced the amount of LDH released compared to the control group (*P* < 0.001) (Fig. 5b). In addition, the LDH release treated with plantaricin RX-8 was significantly lower compared to the other two groups (*P* < 0.001). Furthermore, TEER were used to assess monolayer integrity. Compared to the control group, the cells treated with bacteriocins showed significantly greater TEER (*P* < 0.001) (Fig. 5c). Moreover, there was no significant difference in the TEER between cells treated with three bacteriocins. These findings suggest that bacteriocins may affect the physical barrier of the epithelial cells. To further explore the protective effect of bacteriocins, the count of *L. monocytogenes* at the lower compartment were measured. The count of *L. monocytogenes* crossed the intestinal epithelium was all reduced after treat with three bacteriocins (Fig. 5d). The present results suggested that plantaricin RX-8 had stronger barrier protection against Caco-2 cells than nisin or enterocin Gr17 (*P* < 0.001).

### Effects of bacteriocins on *L. monocytogene*-induced cellular migration

**Fig. 6:**
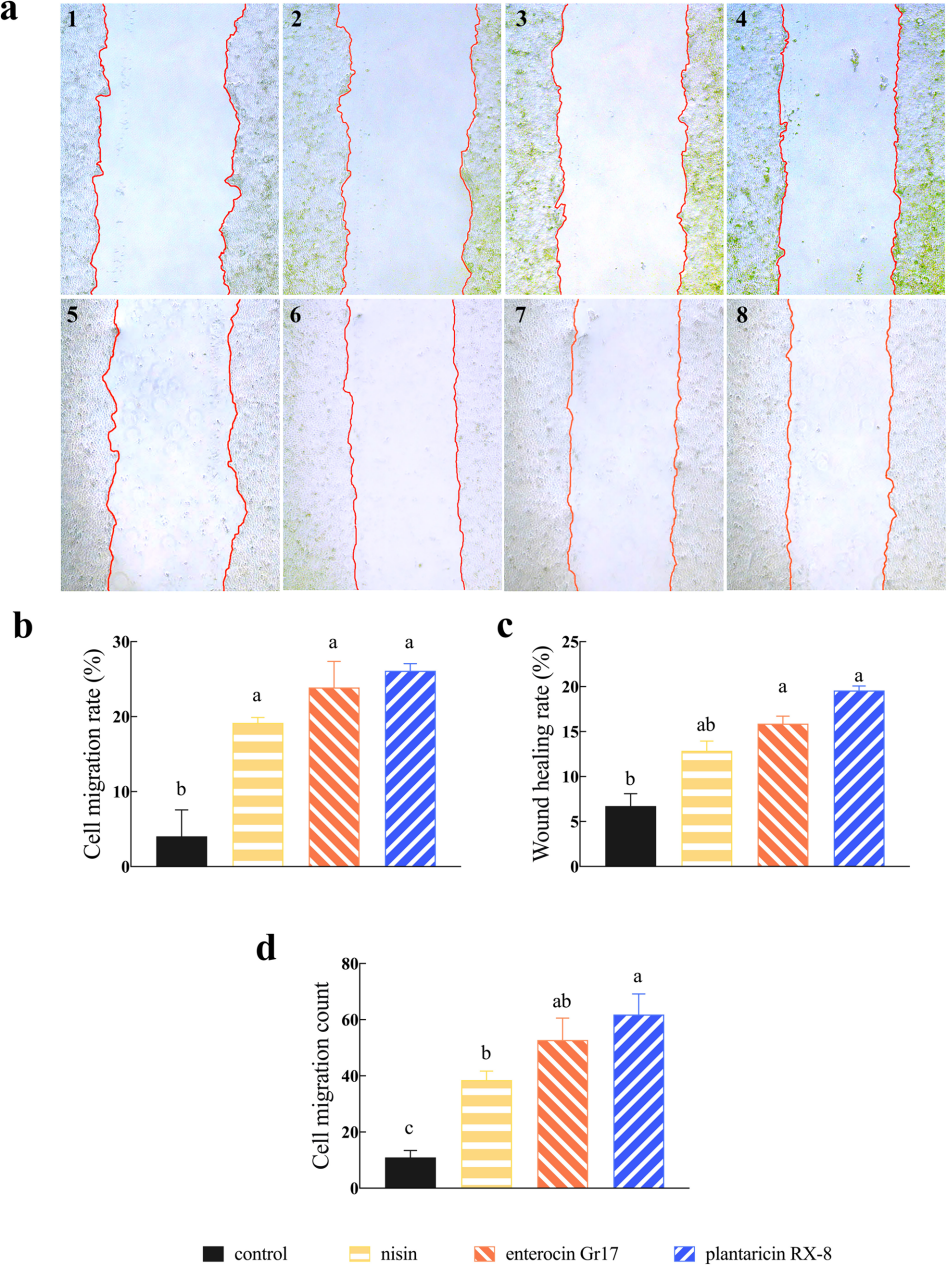
Effect of bacteriocins on *L. monocytogenes*-induced Caco-2 cells mobility. Wound healing assay (**a**), cell migration rate (**b**), wound healing rate (**c**), and cell migration count (**d**)

To clarify the effect of bacteriocins on *L. monocytogenes* induced cell activity, the wound healing area and cell migration counts were determined via transwell assays to determine the migration of Caco-2 cells. Wound healing rate assays were measured on Caco-2 cells induced by *L. monocytogenes*, with and without bacteriocins at 0 and 4 h, respectively. In Fig. 6a, compared to control group, nisin, enterocin Gr17, and plantaricin RX-8 all exhibit certain migration at 4 h. To further quantify the role of bacteriocins on promoting the cellular migration, the cell migration rate (Fig. 6b) and wound healed rate (Fig. 6c) were simultaneous estimated. As expected, there was a notable increase of cell migrations rate in the wound healing assay (*P* < 0.001). Furthermore, transwell assays indicated that cell migration was significantly higher in the plantaricin RX-8 treatment group compared to the other treatment groups (*P* < 0.001) (Fig. 6d). These findings indicated that nisin, enterocin Gr17, and plantaricin RX-8 could attenuate *L. monocytogenes*-induced epithelial physical barrier damage. The effect of bacteriocins could be attributed to the suppression of *L. monocytogenes*-induced Caco-2 cells, which indirectly improves cell activity, rather than acting directly by promoting cell growth and migration.

### Effects of bacteriocins on *L. monocytogenes*-induced tight junction disruption expression

**Fig. 7:**
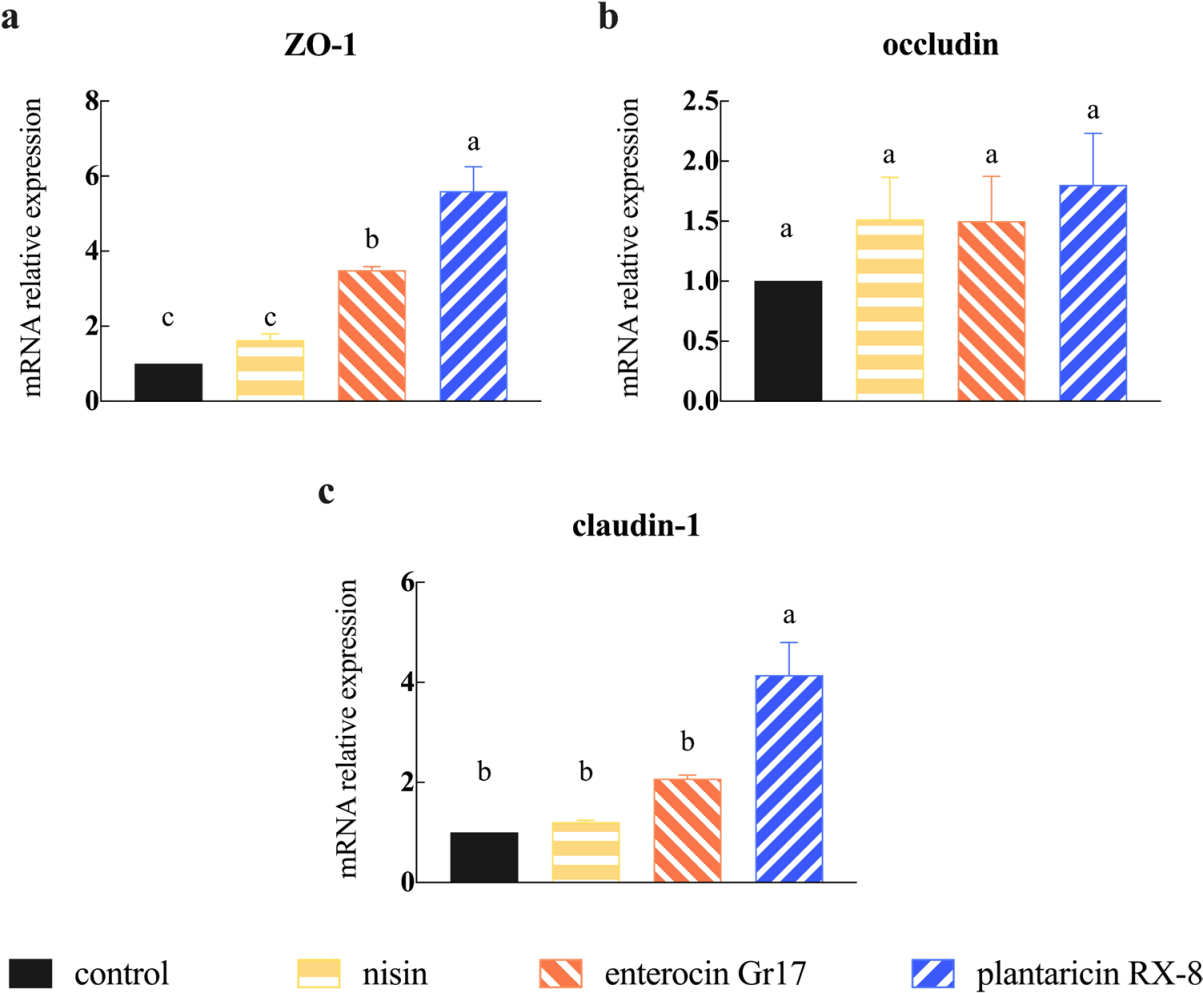
Effect of bacteriocins on the gene expression of ZO-1 (**a**), occludin (**b**), and claudin-1 (**c**) in *L. monocytogenes*-induced Caco-2 cells

The purpose of this study was to investigate the protective effect of bacteriocins on TJP disruption induced by *L. monocytogenes*, specifically focusing on the expression of ZO-1, claudin-1, and occludin. As expected, treatment of bacteriocins significantly upregulated (*P* < 0.001) the relative abundance of mRNA of ZO-1 (Fig. 7a), occludin (Fig. 7b), and claudin-1 (Fig. 7c). Specifically, plantaricin RX-8 treatment group significantly upregulated the gene expression of ZO-1 and claudin-1 compared with other treatment groups.

**Fig. 8:**
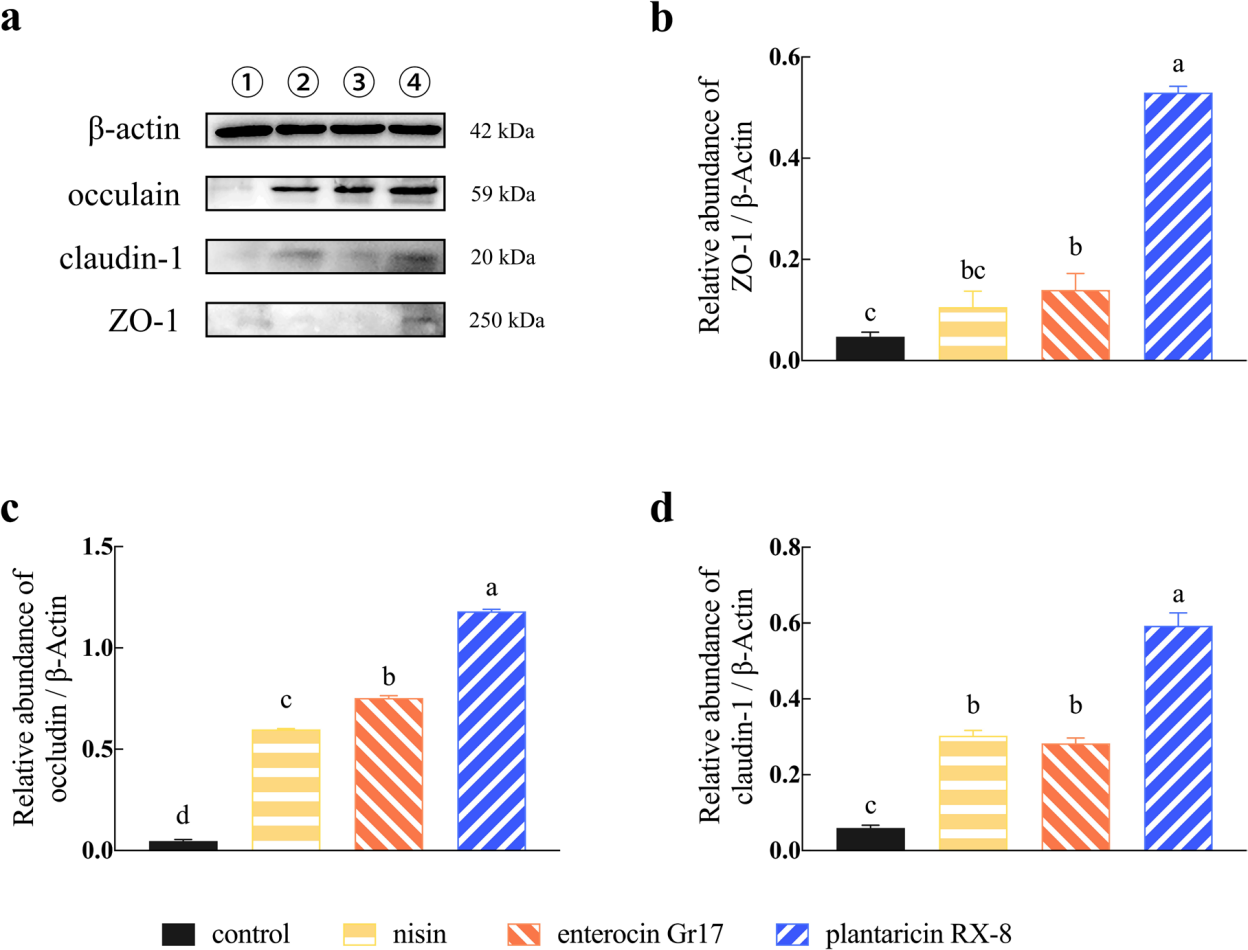
Effects of bacteriocins on intestinal TJP protein expression in *L. monocytogenes*-induced Caco-2 cells. Western blotting bands of TJP (**a**), protein abundance of occludin (**b**), claudin-1 (**c**), and ZO-1 (**d**)

To corroborate mRNA results, the western blotting experiments were employed to detect the protein abundance of the TJP in Caco-2 cells induced by *L. monocytogenes* (Fig. 8a–d). Consistent with the mRNA results, the western blot results demonstrated that the expression of ZO-1, occludin, and claudin-1 was significantly lower in the cells pretreated with bacteriocins (*P* < 0.001). Additionally, three bacteriocins exhibited varying degrees of effectiveness, ranked in descending order as follows: plantaricin RX-8, enterocin Gr17, and nisin. Thus, bacteriocins may attenuate the effect of *L. monocytogenes* on tight junctions in Caco-2 cells.

### Effects of bacteriocins on *L. monocytogenes*-induced inflammation disruption

**Fig. 9:**
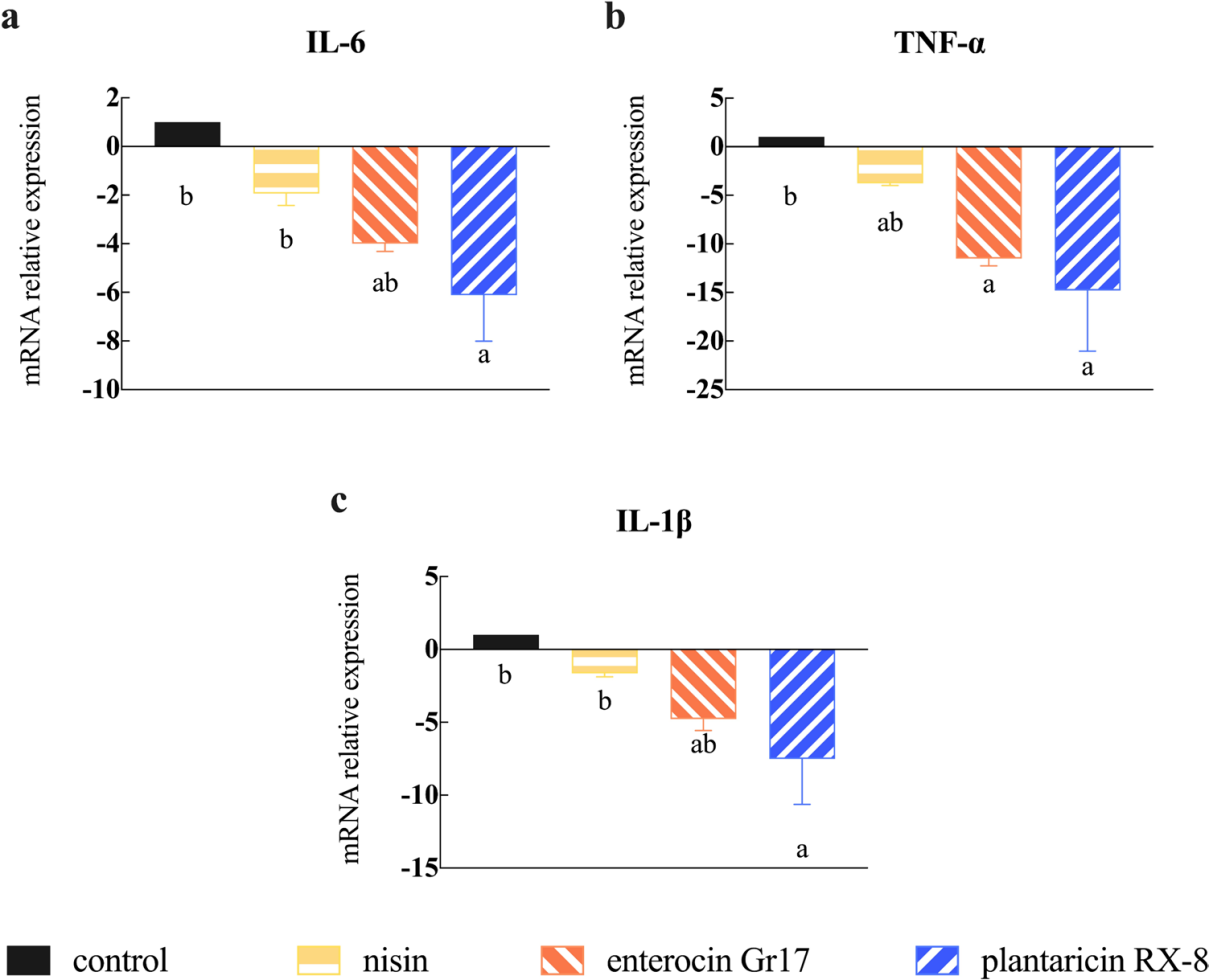
Effects of bacteriocins on gene expression of pro-inflammatory cytokines IL-6 (**a**), TNF-α (**b**), and IL-1β (**c**) transcript levels in Caco-2 cells induced by *L. monocytogenes*

**Fig. 10:**
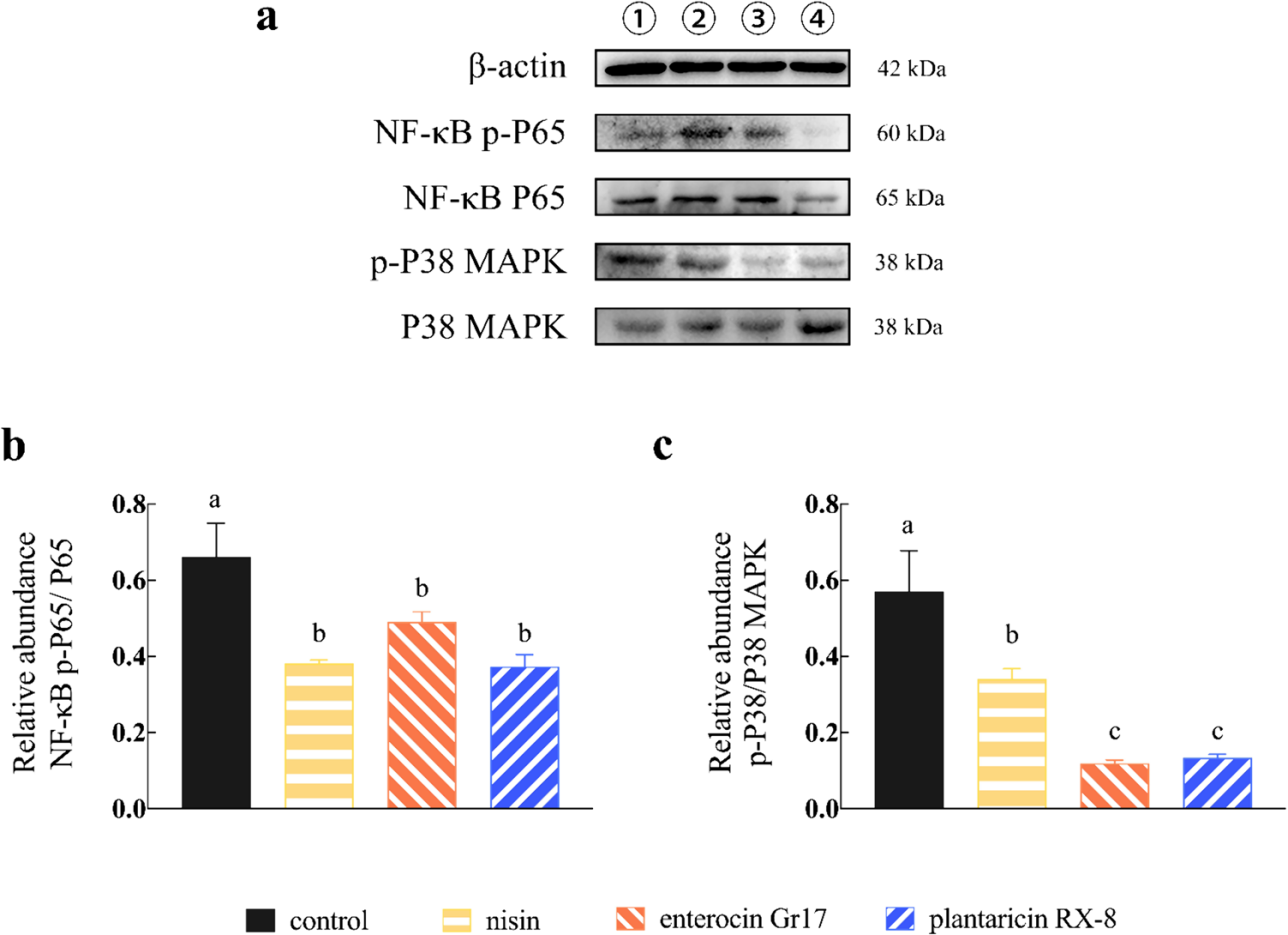
Effects of bacteriocins on immune pathway protein expression in *L. monocytogenes*-induced Caco-2 cells. Western blotting bands of immune pathway protein (**a**), protein abundance of p-P65 (**b**) and p-P38 (**c**)

Before evaluating the effect of bacteriocins on inflammation index induced by *L. monocytogenes*, whether *L. monocytogenes* can induce inflammation was measured (Fig. S3–4). Firstly, the gene expression level of IL-6, TNF-α, and IL-1β were analyzed. The gene expression level in the bacteriocin treatment groups was significantly downregulated compared to the control group when cells were challenged with *L. monocytogenes* (*P* < 0.001) (Fig. 9a–c)*.* After conducting the aforementioned experiments, we preliminarily demonstrated whether bacteriocins can alleviate the inflammation response. This prompted us to examine the interaction between bacteriocins in the nuclear factor kappa B (NF-κB) and mitogen-activated protein kinase (MAPK) pathways, which are crucial cellular cascades involved in inflammation. Bacteriocin-treat groups showed a significant decrease in the abundance of P65 (Fig. 10b) and P38 (Fig. 10c) proteins compared to controls, with no significant differences between bacteriocins (*P* < 0.001). These results prompted us to further confirm the interaction between nisin, enterocin Gr17, and plantaricin RX-8 in the NF-κB and MAPK pathways, which are associated with an inflammatory response.

## Discussion

The aim of this study was to investigate the potential protective effects of three bacteriocins (nisin, enterocin Gr17, and plantaricin RX-8) against *L. monocytogenes*-induced dysfunction of the intestinal barrier in Caco-2 cells. The study findings indicated that three bacteriocins did not exhibit cytotoxicity in the Caco-2 cell line, as determined by CCK-8 assays. Simultaneously, they exhibited potent antimicrobial effect against *L. monocytogenes* and significantly reduced the *L. monocytogenes* adhesion to Caco-2 cells. Furthermore, bacteriocins have shown to effectively attenuate intestinal barrier dysfunction and *L. monocytogenes*-induced inflammatory responses. *L. monocytogenes* is a pathogenic food-borne bacterium that seriously threatens human health. Thus, effective methods must be actively developed to control the spread of pathogen. Bacteriocins are small peptides or complex proteins that are synthesized by ribosomes and have excellent antimicrobial activities. It has been demonstrated that bacteriocins have strong antibacterial activity against pathogenic bacteria *in vitro* (Yi et al. 2018), but whether their inhibitory activity can be maintained in the body is unclear. In addition, the bacteriostatic effect of bacteriocins is mainly due to the structures of different bacteriocins, resulting in differences in their antibacterial mechanisms. However, it is unclear how bacteriocins exhibit antibacterial activity and inhibit pathogenic bacteria and their underlying mechanisms *in vivo*.

In summary, a thorough evaluation of the antibacterial activity of bacteriocins against pathogenic bacteria and their underlying mechanisms *in vivo* is necessary. To address this need, the present study initially evaluated the antibacterial activities of bacteriocins (nisin, enterocin Gr17, and plantaricin RX-8) against *L. monocytogenes in vitro*, respectively. Bacteriocins at concentrations of 1 × MIC, 1/2 × MIC, and 1/4 × MIC were found to be effective in inhibiting the growth of *L. monocytogenes*. Among them, the antibacterial activity of bacteriocins at 1 × MIC on *L. monocytogenes* was significant compared to other concentrations. Thus, bacteriocins were shown to have extremely bactericidal activity against *L. monocytogenes*, which was consistent with previous studies (Yi et al. 2018)*.* Compared to animal studies, *in vitro* cell culture is often the initial step in drug response testing due to its advantages of low cost and ease of handling. In this study, highly similar Caco-2 cells were chosen as a model to investigate the gut barrier function in normal intestinal epithelial cells. Regarding previous studies, bacteriocins are not toxic to cells (Musa et al. 2021). Based on the antibacterial activity and cytotoxicity results, the 1 × MIC (16 μg/mL) was selected for subsequent experiments. Due to the excellent antibacterial ability of three bacteriocins against *L. monocytogenes*, bacteriocins were used to inhibit the adhesion of *L. monocytogenes* in Caco-2 cells. Thus, the present study was conducted in three different settings, as described in Materials and methods. Specifically, in adhesion assays, we observed that bacteriocins can significantly inhibit *L. monocytogenes* adhesion to Caco-2 cells through competition, exclusion, and displacement experiments. Among them, the addition of bacteriocins prior to *L. monocytogenes* was found to be more effective in inhibiting adhesion than attempts to disrupt established colonization. In accordance with previous studies, *Bacillus coagulans* T242 had an inhibitory effect on the adhesion and colonization of intestinal epithelial cells that were inhibited by *Salmonella typhimurium* (Yi et al. 2018). Thus, the ability of bacteriocins to inhibit the adhesion of *L. monocytogenes* in Caco-2 cells was evaluated by exclusion experiments. Furthermore, we hypothesized that bacteriocins repelled *L. monocytogenes* adhesion and invasion by competing for adhesion sites on the surface of Caco-2 cells.

Bacteriocins have excellent antibacterial activity against *L. monocytogenes*, but it could not completely eliminate them, and some *L. monocytogenes* still adhered to the Caco-2 cells surface and invaded the cell interior (Gomes et al. 2012). Therefore, we investigated the viability of *L. monocytogenes* after cell invasion. All three bacteriocins had a weaker antibacterial activity in the intestinal model than *in vitro*, it has also been shown that the bacteriocin MccJ25 similarly inhibits ETEC K88 weaker in IPEC-J2 cell monolayers than *in vitro* (Yu et al. 2018). Furthermore, the number of *L. monocytogenes* in extracellular was higher than that in intracellular, indicating that bacteriocin mainly inhibited the activity of *L. monocytogenes* in extracellular, which was consistent with Gao’s study (2023). *B. coagulans* T242 cell-free supernatants inhibited primarily the adhesion phase of *Salmonella typhimurium* in their study. The varying antibacterial activities of three types of bacteriocins against *L. monocytogenes* may be attributed to their distinct structures. The polycyclic nature of nisin makes them resistant to heat, proteolytic digestion, and have the ability to disrupt the cell membrane integrity of bacteria (Wiedemann et al. 2001). Class IIa bacteriocins have a β-folded structure at the N-terminus connected by a disulfide bond. Research has indicated that the presence of the disulfide bonds can increase the stability of the structure, resulting in a stronger bacteriostatic effect (Kaur et al. 2011). It is important to note that the number of disulfide bonds present impact the bacteriostatic effect (Liu et al. 2019). Class IIb bacteriocins have two peptide chains that form a helix-helix structure. The van der Waals forces and Cα−H…O hydrogen bonds between them contributed the stabilization of the tertiary structure (Ekblad et al. 2016). Meanwhile, class II bacteriocins can act on LuxS/AI-2 in bacteria but not on nisin (Pei et al. 2022).

To clarify how bacteriocins affect barrier function, we analyzed the expression of key virulence factors in *L. monocytogenes* based on previous studies (Wu et al. 2023). In this study, expression levels of *L. monocytogenes* virulence factors were downregulated by nisin, enterocin Gr17, and plantaricin RX-8 in Caco-2 cells. These findings may indicate that the antibacterial ability of bacteriocins may vary depending on the type and chemical composition of the bacteriocin. Class I bacteriocin (nisin) inserts from the N-terminus into the monomolecular layer of the cell membrane of *L. monocytogenes* and interacts with peptidoglycan to form a cage-like structure, which results in the formation of a non-selective pore (Wu et al. 2023). The helical-structure of class II bacteriocin can be adsorbed to the cell membrane through its hydrophobic regions, resulting in a barrel-stave model (Shai 1999). Thus, non- or semi-selective pores are formed in the cell membrane by class II bacteriocins, thereby promoting the death of the target cells. Furthermore, it is worth noting that enterocin Gr17 (class IIa bacteriocin) has a considerable antimicrobial activity. This is due to the N-terminal sequence of xxYGNGVxC (Liu et al. 2019), which binds specifically to the IIC subunit of the *L. monocytogenes* mptACD manipulator (Drider et al. 2006). In addition, plantaricin RX-8 (class IIb bacteriocin) significantly downregulated the transcript levels of *inlB*, *plcA*, *plcA*, and *actA* (*P* < 0.001). Previous research has suggested that peptide chains PlnE and PlnF, which make up the plantaricin RX-8, have bacteriostatic properties individually (Ekblad et al. 2016). Consistent with our study, the inhibitory effect of action of plantaricin YKX (class IIb bacteriocin) on *S. aureus* has been established (Pei et al. 2022). However, the plantaricin RX-8 had no significant impact on the expression of the virulence factor *prfA* (*P* < 0.001), which could be due to the more obvious influence of culture environment on *prfA* expression (Stoll et al. 2008). Similarly, Ake et al. (2011) revealed no positive correlation between *prfA* and other virulence factors regulated by it. Simultaneously, preventing *L. monocytogenes* from entering the cytoplasm and spreading from cell to cell is crucial for intracellular infection. Moreover, it has been reported that *L. monocytogenes* can develop resistance to nisin and class IIa bacteriocins, due to changes in both the phospholipid composition and fatty acid composition of the cell membrane (Kaur et al. 2011). Therefore, plantaricin RX-8 significantly inhibited *L. monocytogenes* invasion in Caco-2 cells (*P* < 0.001).

The invasion of *L. monocytogenes* into cells resulted in increased permeability of Caco-2 cells. It was observed that *L. monocytogenes* was able to pass through the monolayer Caco-2 cell model in large numbers, leading to an increased LDH release and *L. monocytogenes* colony counts in the lower compartment (abdomen compartment). Simultaneously, bacteriocins significantly increased the TEER and indicated a decrease in cell-cell epithelial permeability. In particular, plantaricin RX-8 significantly improved the permeability of Caco-2 cells under invasion by *L. monocytogenes*. Based on the above results, we speculated that bacteriocin can not only directly inhibit the activity of *L. monocytogenes* but can also improve the permeability of epithelial cells. In this experiment, all three bacteriocins increased the cell migration rate and wound healing rate of Caco-2 cells to some extent in the scratch assay, and the number of migrating cells was also significantly increased (*P* < 0.001). These results suggested that bacteriocins enhance the viability of Caco-2 cells, and we hypothesized that the effect is achieved through the inhibition of *L. monocytogenes.* Cell permeability and cell migration are two indicators of cell viability and integrity, which to some extent can reflect the state of cell damage (Wang et al. 2021). Therefore, bacteriocins have been shown to have protective effects on *L. monocytogenes*-induced intestinal barrier disfunction (Ekblad et al. 2016), and the bacteriocins were ranked in descending order according to the following: plantaricin RX-8, enterocin Gr17, and nisin. These results validate the antimicrobial activity of bacteriocins against *L. monocytogenes* in an intestinal epithelial model.

The ameliorative effect of bacteriocins on pathogen-induced cell damage may be related to the expression of TJP in intestinal epithelial cells. The TJP include transmembrane proteins such as claudins and occludins, as well as junctional and blocking cytoplasmic scaffolding proteins such as the zonula occludens (ZO) family of proteins, which have a protective role in maintaining the integrity of the gut barrier (Suzuki 2013). By releasing toxins or reducing the internalization of tight junction proteins, pathogens can disrupt the intestinal barrier (Gao et al. 2023). Bacteriocins can improve the stability of the intestinal barrier by increasing the expression of tight junction proteins. Consistent with these results, we manifested that pretreatment with bacteriocins increased the relative mRNA expression and the protein abundance of ZO-1, occludin, and claudin-1. Interestingly, the protein expression of occludin was not significantly different in the bacteriocin treatment group compared to the control group, which may be due to differences in the source and the mode of action of the peptide. These findings suggested that bacteriocins can protect the integrity of intestinal epithelial by directly inhibiting pathogens and providing a physical barrier between pathogens and intestinal cells.

When pathogenic microorganisms invade cells, immune cells are stimulated and inflammatory factors are activated, resulting in increased damage to the cell barrier. *L. monocytogenes* infection not only damages the intestinal barrier but also affects the immune response of Caco-2 cells. In this study, bacteriocin pretreatment reduced *L. monocytogenes*-induced secretion and gene expression of proinflammatory cytokines, including IL-6, IL-1β, and TNF-α. This may be because *L. monocytogenes* induces epithelial cells to produce proinflammatory cytokines, alters adhesion sites and cytoskeletal recombination, regulates cellular barrier function, and forms an inflammatory micro-environment (Lee et al. 2016). Activation of the NF-κB and MAPK pathways was reported to mediate the expression of pro-inflammatory cytokines (Chen et al. 2020). Earlier studies have also investigated the role of MAPK and NF-κB signaling molecules in the expression of inflammatory cytokine, and links have been established between TNF-α, IL-6 or IL-1β and intestinal permeability. P38, as a stress-related protein kinase, is an important enzyme of MAPK signaling pathway (Falcicchia et al. 2020). The heterodimers formed by P65 are responsible for gene transcription when activating the canonical NF-κB signaling pathway (Zheng et al. 2023), and the activation of canonical NF-κB is closely related to the inflammatory response. The research demonstrated that through downregulating the NF-κB and MAPK pathways induced by *L. monocytogenes*, bacteriocins may inhibit the expression of inflammatory cytokines. Furthermore, three bacteriocins showed similar effects in induced *L. monocytogenes* activation of the NF-κB pathway. Compared with nisin (class I bacteriocin), the activation of the MAPK pathway was significantly inhibited by enterotoxin Gr17 (class IIa bacteriocin) and plantaricin RX-8 (class IIb bacteriocin) (*P* < 0.001). However, enterocin Gr17 and plantaricin RX-8 had significant effects on the MAPK pathway. This may be due to nisin treatment downregulating the levels of IL-6 expression only to a minor extent, which have been reported to induce MAPK activation. Consistent with our study, nisin acted only on the MAPK pathway (Heeney et al. 2019), but enterocin CBT-SL5 (class IIa bacteriocin) and plantaricin BM-1 (class IIb bacteriocin) significantly attenuated the activation of MAPK and NF-κB pathways (Lubelski et al. 2008; Musa et al. 2021). Furthermore, previous studies have demonstrated the correlation between barrier dysfunction and MAPK activation, which may be mediated by pro-inflammatory cytokines (Lee et al. 2016). Therefore, to fully understand the effect of bacteriocins on TJ regulation, it is necessary to investigate other signaling pathways.

Inhibiting food-borne pathogenic bacteria such as *L. monocytogenes*, is essential to improving food safety. There is a growing need for antibiotic substitutes with superior antibacterial activity and non-toxic side effects. Therefore, the study elucidates the inhibitory effect of three bacteriocins (nisin, enterocin Gr17, and plantaricin RX-8) on *L. monocytogenes in vitro* and the protective effects of bacteriocins on intestinal barrier dysfunction and inflammatory responses induced by *L. monocytogenes*, respectively. All three bacteriocins inhibited the growth of *L. monocytogenes* and had no cytotoxicity. Bacteriocins can preempt or compete for *L. monocytogenes* adhesion sites on Caco-2 cells, thereby inhibiting the adhesion and invasion of *L. monocytogenes*. Subsequently, bacteriocin treatment mediated the expression of key virulence factors in *L. monocytogenes*. Indicators such as LDH, TEER, cell mobility, wound healing rate, and cell migration count showed that the presence of bacteriocins effectively inhibited the invasion of *L. monocytogenes* into Caco-2 cells. In addition, bacteriocin may also alleviate the decreased expression of occludin, ZO-1, and claudin-1 proteins in Caco-2 cells caused by *L. monocytogenes* infection, thereby preventing barrier dysfunction induced by *L. monocytogenes*. Bacteriocins were further found to relieve inflammation responses through modulating of IL-6, IL-1β, and TNF-α levels, inhibiting MAPK and NF-κB activation. Among three bacteriocins, plantaricin RX-8 has the best antibacterial activity against *L. monocytogenes* and a protective effect on the intestinal barrier due to its unique structure and inhibitory mechanism. Therefore, these findings indicated that bacteriocin had the potential as an antibiotic substitute. Due to the Caco-2 cell model is insufficient to fully elucidate the inhibitory effect of bacteriocin *in vivo*, further exploration of the attenuation ability and improved effect of bacteriocin on the disruption of intestinal function induced by *L. monocytogenes* in animal models is needed.

## Supplementary information

ESM 1 The following are the supplementary data to this article. (PDF 541 kb)

## Data Availability

The data in this study are all presented in the article.
