# Supplementary material for: Bacteriocins attenuate Listeria monocytogenes–induced intestinal barrier dysfunction and inflammatory response
Source: Appl Microbiol Biotechnol. 2024 Jun 19;108(1):384. doi: 10.1007/s00253-024-13228-w (PMC11186933; doi:10.1007/s00253-024-13228-w)
Supplement: Supplementary file 1 — The following are the supplementary data to this article. (PDF 541 kb) [file 253_2024_13228_MOESM1_ESM.pdf]

Journal name: Applied Microbiology and Biotechnology

Manuscript title: Bacteriocins Attenuate *Listeria monocytogenes* Induced Intestinal Barrier Dysfunction and Inflammatory Response

Authors: Zhao Wang[†, 1], Jing Du[†, 1], Wenyu Ma[1], Xinjie Diao[1], Qi Liu[1], Guorong Liu[1, 2, 3, *]

Affiliation: [1] School of Food and Health, Beijing Technology and Business University, Beijing 100048

[2] Key Laboratory of Geriatric Nutrition and Health, Ministry of Education, Beijing Technology and Business University, Beijing 100048

[3] Beijing Engineering and Technology Research Center of Food Additives, Beijing Technology and Business University, Beijing 100048

[†] Zhao Wang and Jing Du contributed equally to the manuscript

Corresponding authors: Guorong Liu[1, 2, 3, *] Tel: 00861068985456. Email: liuguorong@th.btbu.edu.cn

Postal address: Room 303, No.8 Building, East campus of Beijing Technology and Business University, No.11 of Fucheng Road, Haidian District, Beijing 100048, China

Supplementary Tables

Table. S1 List of primer sequences

Gene	Primer sequence	Source
RT-*β-actin*-F	GGCTGTATTCCCCTCCATCG	This study
RT-*β-actin*-R	CCAGTTGGTAACAATGCCATGT	This study
RT-*IL-6*-F	GAGAAAGGAGACATGTAACAAGAG	(Guo et al. 2022)
RT-*IL-6*-R	GCTCTGGCTTGTTCCTCAC	(Guo et al. 2022)
RT-*IL-1β*-F	GTGGCAATGAGGATGACTTGTTC	(Guo et al. 2022)
RT-*IL-1β*-R	TTGCTGTAGTGGTCGGAG	(Guo et al. 2022)
RT-*TNF-α*-F	GGCAGTCAGATCATCTTCTCGAAC	(Guo et al. 2022)
RT-*TNF-α*-R	TGGTAGGAGACGGCGATGC	(Guo et al. 2022)
RT-*ZO-1*-F	TGATGGTGTCCTACCTAATTCAACTCA	(Peng et al. 2022)
RT-*ZO-1*-R	GAACGCCAGCTACAAATATTCCAACA	(Peng et al. 2022)
RT-*claudin-1*-F	AGAACAGAGCAAGATCACTATGAGACA	(Peng et al. 2022)
RT-*claudin-1*-R	CTTTGTTGATCTGAAGTGATAGGTGGA	(Peng et al. 2022)
RT-*occludin*-F	GCACATACCTTCATGTGGCTCAG	(Peng et al. 2022)
RT-*occludin*-R	TGGAACAGAGCACAAACATGTCA	(Peng et al. 2022)
RT-*inlA*-F	GAATGTAACAGACACGGTCTCAC	(Qiao et al. 2022)
RT-*inlA*-R	TCCCTAATCTATCCGCCTGAAG	(Qiao et al. 2022)
RT-*inlB*-F	CGAAAGTACAAGCGGAGACTATC	(Qiao et al. 2022)
RT-*inlB*-R	GTTTCTGCAAAAGCATCATCTG	(Qiao et al. 2022)
RT-*actA*-F	TAGCGTATCACGAGGAGG	(Qiao et al. 2022)
RT-*actA*-R	TTTTGAATTTCATATCATTCACC	(Qiao et al. 2022)
RT-*prfA*-F	ATGAACGCTCAAGCAGAAG	(Qiao et al. 2022)
RT-*prfA*-R	GTTTTGGTTTTATCCCGTTAGTT	(Qiao et al. 2022)
RT-*plcA*-F	TCGGACCATTGTAGTCATCTTG	(Qiao et al. 2022)
RT-*plcA*-R	TCACGCAAATTCGGCATGC	(Qiao et al. 2022)
RT-*plcB*-F	CGCAGCTCCGCATGATATT	(Qiao et al. 2022)
RT-*plcB*-R	TTATCCGCGGACCAACTAAG	(Qiao et al. 2022)

Table S2 MIC of bacteriocins against *L. monocytogenes*

**bacteriocin**	**nisin**	**enterocin Gr17**	**plantaricin RX-8**
MIC (μg/mL)	16	16	16

Table S3 The scientific names list

Abbreviation	Full name
Caco-2 cells	Human colorectal adenocarcinoma cells
CCK-8	Cell counting kit-8
DMEM	Dulbecco's modified eagle medium
IL-6	Interleukin-6
IL-1β	Interleukin-1 beta
LDH	Lactate dehydrogenase
MAPK	Mitogen-activated protein kinases
MRS	De Man, Rogosa and Sharpe broth
MIC	Minimum inhibitory concentration
NF-κB P65	Nuclear factor kappa B P65
NF-κB p-P65	Nuclear factor kappa B phosphorylated P65
P38 MAPK	P38 Mitogen-activated protein kinase
p-P38 MAPK	phosphorylated P38 Mitogen-activated protein kinase
PBS	Phosphate buffered saline
RT-qPCR	Quantitative real-time polymerase chain reaction
TBST	Tris-buffered saline with tween 20
TEER	Trans-epithelial electrical resistance
TJP	Tight junction proteins
TNF-α	Tumor necrosis factor-alpha
TSA	Tryptic soy agar
TSB	Trypticase soy broth
ZO-1	Zonula occludens-1

Supplementary Figures

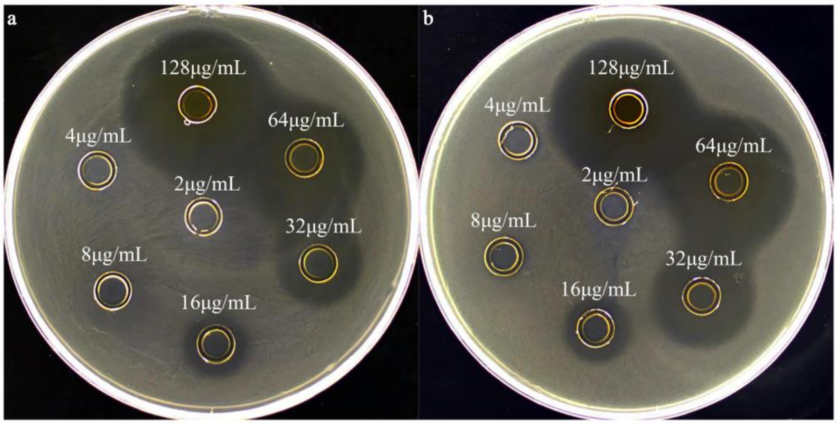

Figure S1 Inhibition zone assay of *L. monocytogenes* treated with enterocin Gr17 (a) and plantaricin RX-8 (b) at different concentrations

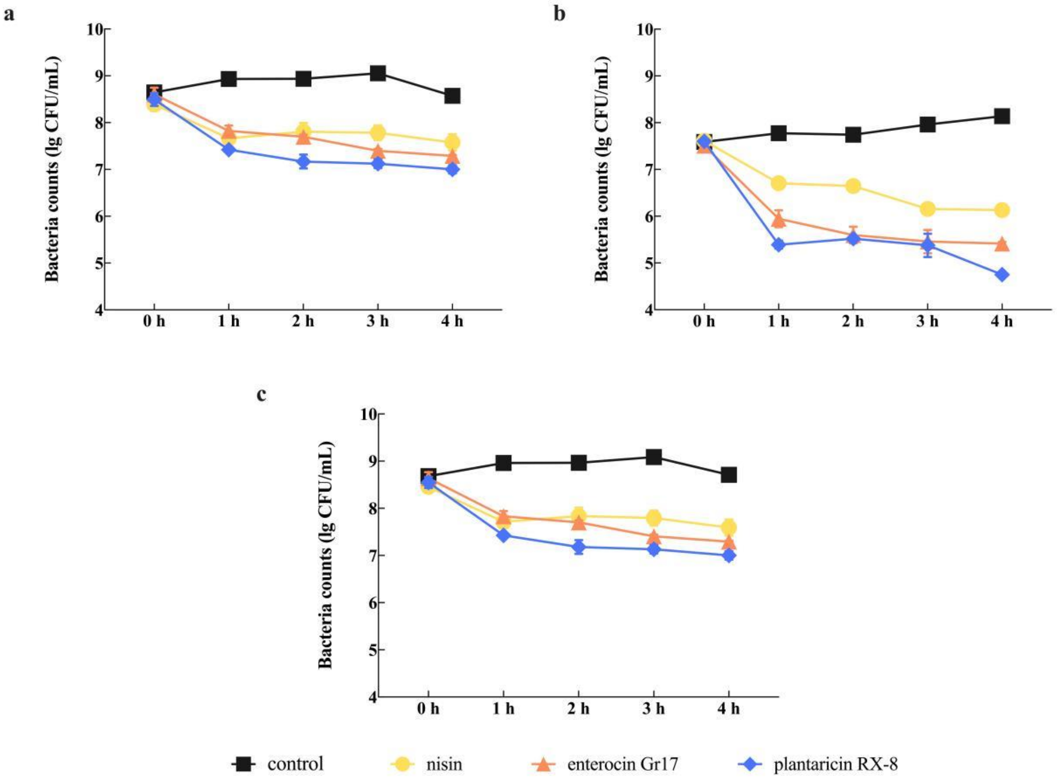

Figure S2 Protective effects of bacteriocins against *L. monocytogenes* infecting Caco-2 cells. *L. monocytogenes* amount at extracellular (a), intracellular (b), and both extracellular and intracellular (c)

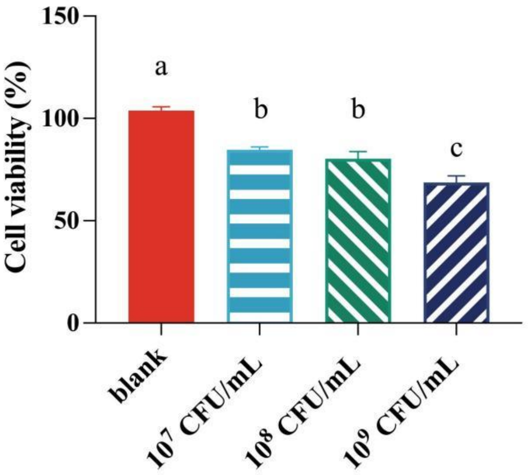

Fig S3 Cell cytotoxicity assays of *L. monocytogenes* in Caco-2 cells

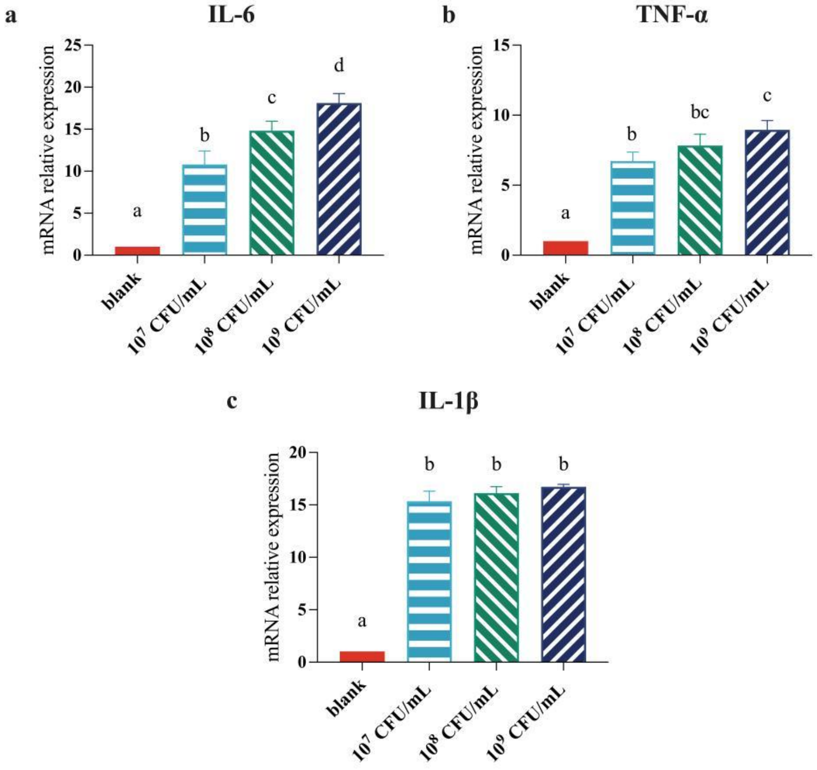

Fig S4 Gene expression levels of pro-inflammatory cytokines IL-6 (a), TNF-α (b), and IL-1β (c) transcript levels in Caco-2 cells induced by *L. monocytogenes*

References

Guo, D, Bai, F, Zhan, X, Zhang, W, Jin, T, Wang, Y, Xia, X, & Shi, C (2022) Citral mitigates inflammation of Caco-2 cells induced by *Cronobacter sakazakii*. Food Funct, 13(6), 3540-3550. http://dx.doi.org/10.1039/d2fo00098a

Peng, B, Cui, Q, Ma, C, Yi, H, Gong, P, Lin, K, Liu, T, & Zhang, L (2022) *Lactiplantibacillus plantarum* YZX28 alleviated intestinal barrier dysfunction induced by enterotoxigenic *Escherichia coli* via inhibiting its virulence factor production. Food Bioscience, 50. http://dx.doi.org/10.1016/j.fbio.2022.102050

Qiao, Z, Zhang, L, Wang, X, Liu, B, Shan, Y, Yi, Y, Zhou, Y, & Lu, X (2022) Antibiofilm effects of bacteriocin BMP32r on *Listeria monocytogenes*. Probiotics Antimicrob Proteins, 14(6), 1067-1076. http://dx.doi.org/10.1007/s12602-021-09863-8
